# Supplementary material for: ATF-4 and hydrogen sulfide signalling mediate longevity in response to inhibition of translation or mTORC1
Source: Nat Commun. 2022 Feb 18;13:967. doi: 10.1038/s41467-022-28599-9 (PMC8857226; doi:10.1038/s41467-022-28599-9)
Supplement: Supplementary file 1 — Supplementary Information [file 41467_2022_28599_MOESM1_ESM.pdf]

SUPPLEMENTARY INFORMATION

ATF-4 and hydrogen sulfide signalling mediate longevity in response to inhibition of translation or mTORC1

Cyril Statzer[1§], Jin Meng[2,3,4§], Richard Venz[1], Monet Bland[2,3,4], Stacey Robida-Stubbs[2,3,4], Krina Patel[2,3,4], Dunja Petrovic[5], Raffaella Emsley[6], Pengpeng Liu[7], Ianessa Morantte[8], Cole Haynes[7], William B. Mair[8], Alban Longchamp[6], Milos R. Filipovic[5], T. Keith Blackwell[2,3,4*], and Collin Y. Ewald[1*]

1 Eidgenössische Technische Hochschule Zürich, Department of Health Sciences and Technology, Institute of Translational Medicine, Schwerzenbach, Switzerland

2 Department of Genetics, Harvard Medical School, Boston, MA, United States

3 Joslin Diabetes Center, Research Division, Boston, MA, United States

4 Harvard Stem Cell Institute, Cambridge, MA, United States

5 Leibniz-Institut für Analytische Wissenschaften-ISAS-e.V., Dortmund, Germany

6 Department of Vascular Surgery, Centre Hospitalier Universitaire Vaudois and University of Lausanne, Lausanne, Switzerland

7 Department of Molecular, Cell and Cancer Biology, University of Massachusetts Medical School, Worcester, MA, U.S.A.

8 Department of Genetics and Complex Diseases, Harvard School of Public Health, 665 Huntington Avenue, Boston, MA, U.S.A.

§ Authors contributed equally

*Corresponding authors: collin-ewald@ethz.ch (C.Y.E.) and keith.blackwell@joslin.harvard.edu (T.K.B.). These authors jointly supervised the work.

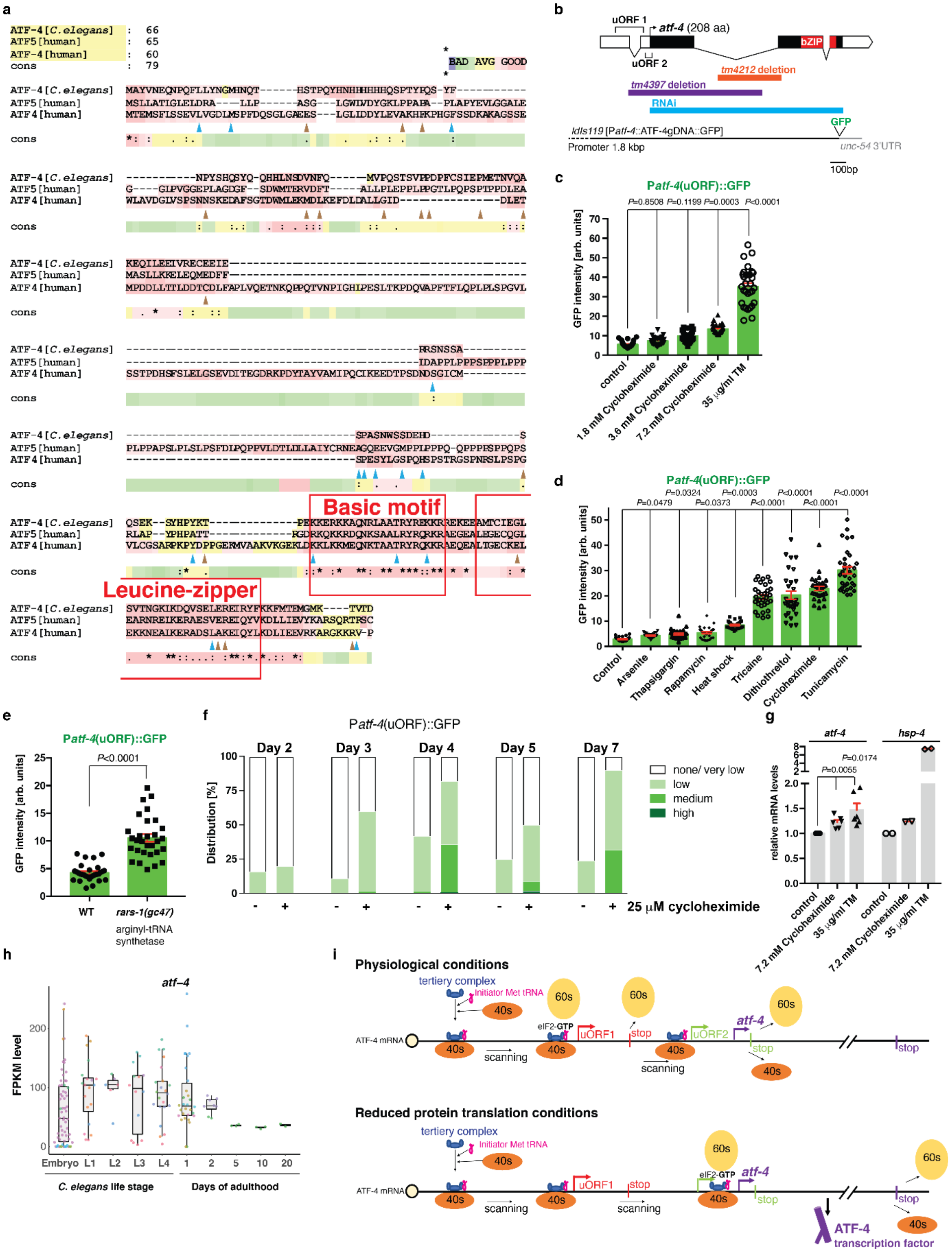

Supplementary Figure 1. Translational regulation of *C. elegans atf-4* expression. a *C. elegans* ATF-4 (T04C10.4) shares identical amino acids with human ATF4 (blue arrowheads) and ATF5 (brown arrowheads), and its basic DNA

binding region is more similar to that of human ATF4. Stars indicate identical amino acids among *C. elegans* ATF-4, human ATF4, and ATF5 (25 in total). Single dots indicate that size or hydropathy is conserved, while double dots indicate that both size and hydropathy are conserved between the corresponding residues. The *C. elegans* ortholog of human ATF5 is ATFS1[1]. **b** Diagram of *atf-4* mRNA, mutations and RNAi clone, and the *Patf-4(uORF)::GFP* transgene. The *atf-4* mRNA has an extensive 5'UTR of 250 nucleotides containing two uORFs, of which uORF1 translates into a 39 amino acids (aa) peptide and uORF2 into a 14 aa peptide. The *tm4397* variation is an 806 base pair (bp) deletion that covers part of uORF1, uORF2, the translational start site and the first exon, suggesting that *tm4397* is a putative null allele. Our genetic analyses aside from *atf-4* RNAi would therefore affect expression of the small ORFs that are imbedded within the 5'UTR. Although such microproteins can have functions[2], considering the totality of evidence reported here, including the evolutionary conservation of ATF-4 regulation and functions, we conclude that it is unlikely that these small ORFs have substantially affected our results. **c** Quantification of GFP fluorescence in P*atf-4*(uORF)::GFP transgenic animals at the L4 stage treated either with cycloheximide for 1 hour or TM for 4 hours. Mean $\pm$ SEM. One-way ANOVA with post hoc Tukey. n > 3 biological replicates. **d** Quantification of GFP fluorescence showing the effects on ATF-4 expression of various drug treatments or interventions that reduce mRNA translation. L4 stage animals were treated either with 20 mM arsenite (an inducer of oxidative stress) for 30 min, 200 mM thapsigargin (which induces ER stress by inhibiting the ER Ca^{2+} ATPase) for 4 hours, 100 μM rapamycin (an inhibitor of mTORC1) for overnight, heat shock at 35°C for 30 min, 2% tricaine for 1 hour (which induces ER stress), 10 mM dithiothreitol (which induces reductive ER stress) for 4 hours, 10 mM cycloheximide (an inhibitor of translation elongation) for 1 hour, or 35 μg/ml tunicamycin (a glycosylation inhibitor that induces ER stress) for 4

hours. Mean ± SEM. One-way ANOVA with post hoc Tukey. n > 3 biological replicates. **e** Nonsense mutation in the arginyl-tRNA synthetase *rars-1(gc47)* increased P*atf-4*(uORF)::GFP expression compared to WT at the L4 stage. Mean ± SEM. Unpaired two-tailed Student's *t*-test, hypothetical mean of 1. **f** Quantification of GFP intensity in transgenic P*atf-4*(uORF)::GFP animals at different ages showing that 25 uM cycloheximide induced ATF-4 induction. n=2 independent trials. L4 animals were transferred onto plates containing either DMSO or cyclohexmide with FUdR. **g** Quantification of *atf-4* mRNA levels after cycloheximide or TM treatment in L4 stage animals. n=3 independent trials, measured in duplicates. In one trial, *hsp-4* mRNA was assessed as a positive control for ER stress. Mean ± SEM. *P* values relative to control determined by one-sample *t*-test, two-tailed, a hypothetical mean of 1. **h** Expression levels of *atf-4* mRNA plotted as Fragments Per Kilobase of transcript per Million mapped reads (FPKM) during development and ageing. The *atf-4* mRNA expression levels of untreated WT *C. elegans* were retrieved using the RNAseq FPKM Gene Search tool (www.wormbase.org). The boxplots represent the overall expression pattern and the color of the individual dots refer to the 32 individual studies used. **i** Hypothetical working model for *C. elegans atf-4* preferential translation, assuming that its regulation is the same as mammalian *ATF4*[3,4]. The *C. elegans atf-4* gene encodes two uORFs. After translating the first uORF, the small ribosomal subunit will continue scanning along the ATF4 mRNA. Under non-stressed conditions, *i.e.*, when high amounts of the eIF2-GFP bound Met-tRNA$_i^{Met}$ are available, the small ribosomal subunit will readily acquire the eIF2 ternary complex, and the large ribosomal subunit will associate to translate the second uORF. In mammalian ATF4, the ribosome disassociates from the *atf-4* mRNA after translating the last uORF. However, under stress or reduced translational conditions, *i.e.,* low amounts of the eIF2-GFP bound Met-tRNA$_i^{Met}$ availability, the association of the

large to the small ribosomal subunit is delayed, whereby the inhibitory second uORF is skipped and the re-initiation complex starts to translate the ATF-4 coding region. Phosphorylation of eIF2α subunit inhibits the guanine nucleotide exchange factor eIF2B, which lowers the exchange of the eIF2-GDP to eIF2-GTP and thereby lowers global mRNA translation initiation.

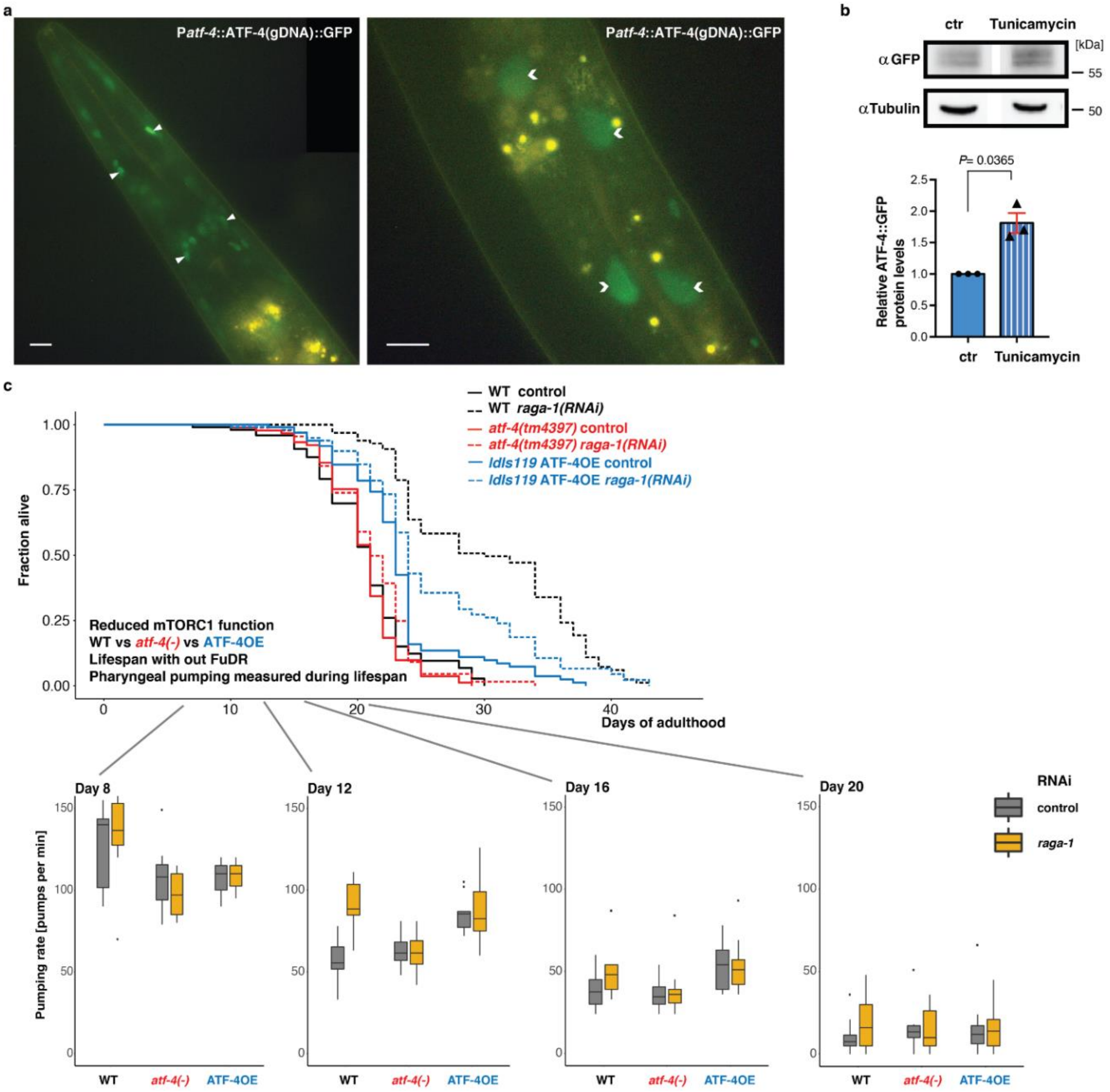

Supplementary Figure 2. Overexpression of ATF-4 increases healthspan. a Representative images showing the expression of ATF-4 in the head (left) and mid-body (right). ATF-4::GFP (*ldIs119*) is displayed in green and found predominantly in nuclei (nuclei of head neurons or glia indicated by arrowheads, intestinal nuclei indicated by chevrons). Yellow puncta are autofluorescent gut granules. 100 x magnification. Scale bar = 10 μm. n > 60 animals from 3 biological replicates. **b** Western blots and quantification showing ATF-4::GFP levels in day-1-adult transgenic ATF-4OE(*ldIs119*) either treated with DMSO (ctr) or 35 μg/mL tunicamycin for 6 hours. n= 3 replicates. Mean ± SEM. One-sample *t*-test, two-

tailed, a hypothetical mean of 1. **c** Pharyngeal pumping measurements across the lifespan comparing WT (N2), *atf-4(tm4397)* mutants, and ATF-4OE(*ldIs119*) treated with either empty vector control RNAi (L4440) or *raga-1(RNAi)*, on plates that do not contain FUdR*.* See Supplementary Table 2 for raw data.

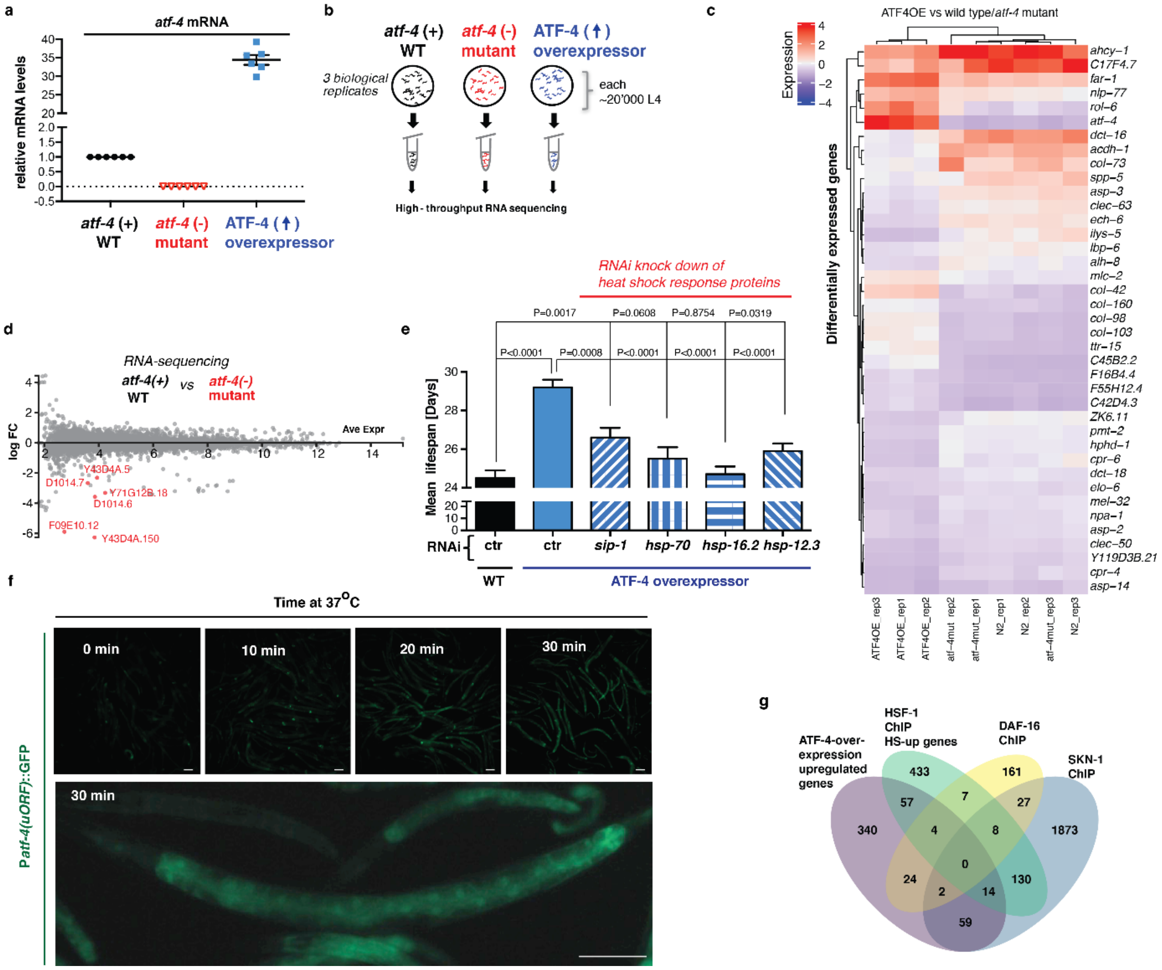

Supplementary Figure 3. RNA-sequencing reveals transcriptional targets of ATF-4. a Quantification of *atf-4* mRNA expression levels in *atf-4(tm4397)* mutants (*atf-4*(-)) and ATF-4OE(*ldIs119*) relative to wild type (*atf-4*(+) WT) animals by qRT-PCR. The same samples were used for RNA sequencing. n=3 independent biological replicates of about 20, 000 L4 *C. elegans*. *P* values for both *atf-4(tm4397)* or ATF-4OE(*ldIs119*) are <0.0001 relative to WT determined by one-sample *t*-test, two-tailed, a hypothetical mean of 1. **b** Schematic representation of sample collection for RNA sequencing. See Methods for details. **c** Hierarchical clustering heatmap of the genes that are most differentially regulated in either direction when comparing ATF-4OE(*ldIs119*) to WT and *atf-4(tm4397)* mutants (*atf-4* (-) mutant). As expected, *atf-4* is in the top gene set. The collagen *rol-6* is the co-injection

marker for the transgenic *ldIs119*. Independent biological replicates are indicated as “rep#”. For details and raw data see Supplementary Table 3. **d** MA (log ratio and mean average)-plot of RNA sequencing analysis comparing *atf-4(tm4397)* mutants (*atf-4* (-) mutant) to absolute log fold-change (FC) relative to WT. In red, highlighted genes with a false discovery rate (FDR) < 0.1 and abs log FC > 1 to WT. Details in Supplementary Table 3. **e** Longevity conferred by ATF-4 overexpression (*ldIs119*) is blunted by knockdown of *sip-1, hsp-70, hsp-16.2,* or *hsp-12.3*. Mean $\pm$ SEM. P values are relative to WT on empty vector RNAi (L4440). For statistical details see Supplementary Table 1. **f** Representative images that heat increases P*atf-4(uORF)*::GFP transgene expression. Bottom panel shows higher magnification. Anterior to the right, ventral side down. Scale bar = 100 μm. **g** Venn diagram showing the overlap of ATF-4 overexpression-upregulated genes with genes that were bound directly by SKN-1, DAF-16, and HSF-1 in chromatin immunoprecipitation (ChIP) studies. For details and references see Supplementary Table 5.

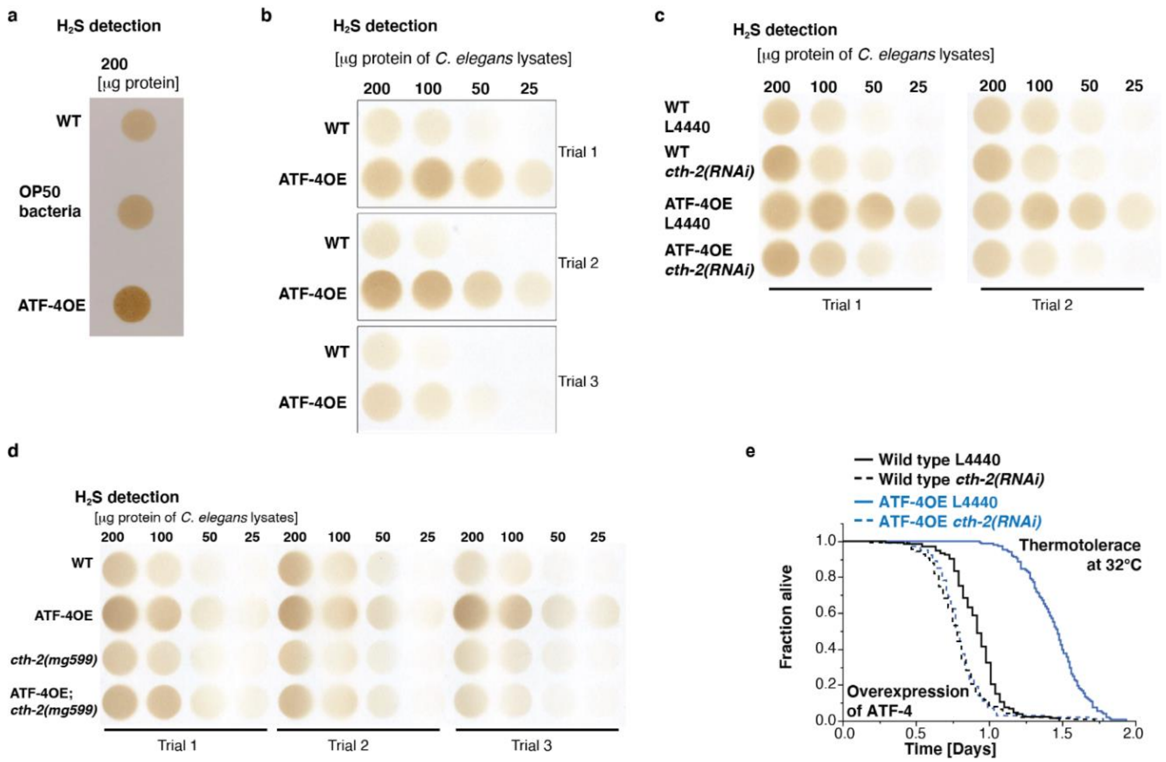

Supplementary Figure 4. ATF-4 overexpression increases H_2S production via CTH-2. a Measurements of H_2S production capacity of protein lysates from WT *C. elegans*, the food source OP50 *E. coli* bacteria alone, or ATF-4OE(*ldIs119*) . Because OP50 lysates have the capacity to produce H_2S, we washed *C. elegans* at least three times or until no bacteria were visible before lysing worms. **b** ATF-4OE(*ldIs119)* exhibited increased H_2S production capacity compared to WT. n=3 independent biological trials. **c** The increase in H_2S production capacity in ATF-4OE was reduced by *cth-2* knockdown. **d** Additional trials of Fig. 4f. **e** The increased heat stress resistance deriving from ATF-4 overexpression was suppressed by *cth-2* knockdown. For H_2S quantification in (**a**)-(**d**), see Supplementary Table 12. For statistical details and additional trials in (**e**) see Supplementary Table 7.

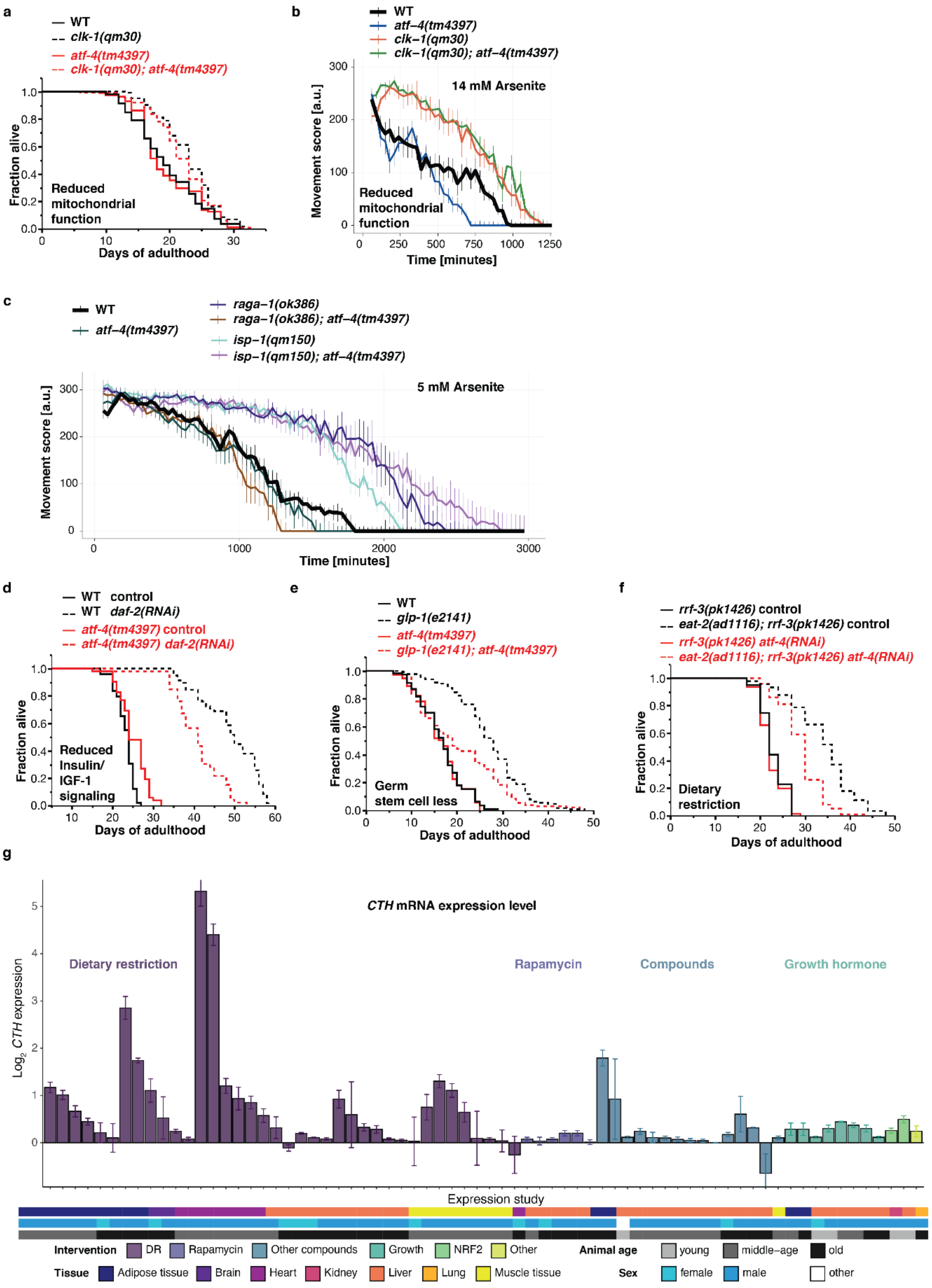

Supplementary Figure 5. A partial role for ATF-4 in various lifespan extension programs. a The longevity of *clk-1(qm30)* animals (impaired mitochondrial function) does not depend on *atf-4*. **b** The increased oxidative stress resistance of

clk-1(qm30) mutants does not require *atf-4*. **c** Loss of *atf-4* suppresses the oxidative stress resistance of *raga-1(ok386)* mutants (reduced mTORC1 activity), but not that of reduced mitochondrial function mutant *isp-1(qm150)*. **d** Longevity arising from reduced insulin/IGF-1 signalling by adult-specific knockdown of the *daf-2* receptor is partially suppressed by *atf-4(tm4397)* mutation. **e** The *atf-4(tm4397)* mutation partially suppresses the longevity of *glp-1(e2141)* mutants (genetic germline stem cell ablation). **f** Knockdown of *atf-4* partially suppresses the longevity of the genetic DR-related model *eat-2(ad1116)* in the RNAi-sensitized *rrf-3(pk1426)* background. **g** CTH mRNA expression levels in long-lived over control mice, analyzed from publicly available expression datasets (Supplementary Table 11). Data are grouped and colored by interventions and represented as Mean $\pm$ SEM. n > 3 biological samples. The meta data of the samples are summarized by colored tiles indicating first the tissue of origin, followed by the sex and then the age group of the mice in each experiment. Animals sacrificed before 16 weeks of age were classified as “young”, between 16 to 32 weeks as “middle-aged” and animals above 32 weeks as “old”. If no meta information could be found, it was labelled as “not specified”. For statistical details and additional trials in (**a**), (**d**)-(**f**), see Supplementary Table 1. For statistical details and additional trials in (**b**), (**c**), and see Supplementary Tables 8.

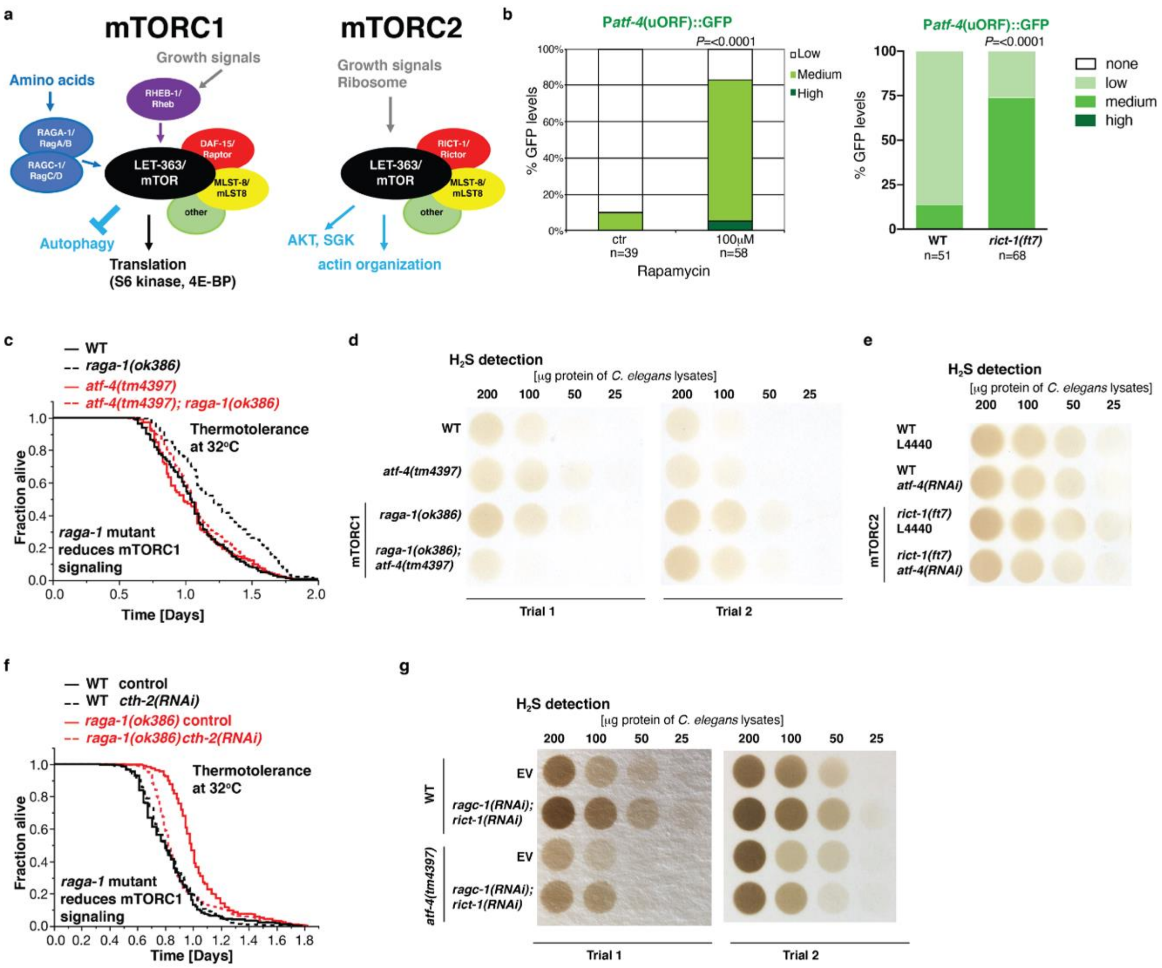

Supplementary Figure 6. Preferential *atf-4* translation and H_2S signalling upon reduced mTOR signalling. a Schematic diagram showing the composition, regulation, and functions of the two mTOR complexes (mTORC1 and mTORC2) adapted from[5]. **b** Rapamycin treatment (left) or *rict-1(ft7) mutation* (right) leads to preferential translation of ATF-4. Rapamycin treatment was initiated at L4. GFP intensity was scored at day 3 of adulthood. *P* values were determined by Chi^2 test. **c** Increased heat stress resistance (32°C) of *raga-1(ok386)* mutants depends on *atf-4*. **d** Two additional independent biological trials of Fig. 7a. **e** One additional independent biological trial of Fig. 7b. **f** Increased heat stress resistance (32°C) of *raga-1(ok386)* mutants depends on *cth-2*. **g** H_2S production capacity assay from *C. elegans* lysates showing that simultaneous knockdown of both *raga-1* and *rict-*

1 starting at L4 increases H_2S production capacity in an *atf-4*-independent manner of day-3 adults. For statistical details and additional trials in (**c**) and (**f**), see Supplementary Table 7; Quantification of H_2S assays in (**d**), (**e**), and (**g**) are in Supplementary Table 12.

Supplementary Table 1: *C. elegans* strains used in this study

Strain	Source	Genotype
N2 Bristol	PMID: 28085666	*wild-type*
LD1325	This paper	*atf-4(tm4397) X*, 7x outcrossed
WBM26	This paper	*wbmEx26* [pWM48 (P*atf-4*::ATF-4(gDNA)::GFP::*unc-54*(3'UTR), pRF4 (*rol-6(su1006)*)] extrachromosomal
WBM27	This paper	*wbmEx27* [pWM48 (P*atf-4*::ATF-4(gDNA)::GFP::*unc-54*(3'UTR), pRF4 (*rol-6(su1006)*)] extrachromosomal
LSD139	This paper	*ldIs119* [pWM48 (P*atf-4*::ATF-4(gDNA)::GFP::*unc-54*(3'UTR), pRF4 (*rol-6(su1006)*)] Integrated from *wbmEx26*, 8x outcrossed
LSD138	This paper	*ldIs120* [pWM48 (P*atf-4*::ATF-4(gDNA)::GFP::*unc-54*(3'UTR), pRF4 (*rol-6(su1006)*)] Integrated from *wbmEx27*, 10x outcrossed
LD1499	PMID: 32958476	[P*atf-4*(uORF)::GFP::*unc-54*(3'UTR)] Integrated, 4x outcrossed
ZD1866	PMID: 28292919	*eif-2α(qd338) I*

LSD2095	This paper	*eif-2α(qd338) I;* [P*atf-4*(uORF)::GFP:: *unc-54*(3'UTR)]
VC2569	PMID: 23173093	*cth-1(ok3319) V*
GR2257	PMID: 30911177	*cth-2(mg599) II*
LSD2146	This paper	*cth-2(mg599) II; ldIs119* [pWM48 (P*atf-4*::ATF-4(gDNA)::GFP::*unc-54*(3'UTR), pRF4 (*rol-6(su1006)*)]
MQ130	PMID:8638122	*clk-1(qm30) III*
LSD2040	This paper	*clk-1(qm30) III; atf-4(tm4397) X*
CB1370	PMID: 9252323	*daf-2(e1370)*
CB4037	PMID: 1457827	*glp-1(e2141) III*
LSD2039	This paper	*glp-1(e2141) III; atf-4(tm4397) X*
MQ887	PMID: 11709184	*isp-1(qm150) IV*
LSD2041	This paper	*isp-1(qm150) IV; atf-4(tm4397) X*
VC222	PMID:23173093	*raga-1(ok386) II*
LSD260	This paper	*raga-1(ok386) II; atf-4(tm4397) X*
LSD2038	This paper	*rars-1(gc47)* III; [P*atf-4*(uORF)::GFP:: *unc-54*(3'UTR)]
KQ1366	A gift from Sengupta lab	*rict-1(ft7) II*
LSD2049	This paper	*rict-1(ft7) II;* [P*atf-4*(uORF)::GFP:: *unc-54*(3'UTR)]
TJ1060	PMID: 7753635	*spe-9(hc88) I; rrf-3(b26) II.*
NL2099	PMID: 11719187	*rrf-3(pk1426) II*
CF1850	PMID: 18282106	*rrf-3(pk1426) II; eat-2(ad1116) II*

Supplementary Table 2: List of oligonucleotides used in this study

atf-4 (qRT-PCR) forward	TCCAAATGGTTCCACAAAGC
atf-4 (qRT-PCR) reverse	GCGCACGATTTCTTCGAG
hsp-4 (qRT-PCR) forward	CACTCCTTCTTTGCGACG
hsp-4 (qRT-PCR) reverse	GAGTAGAAGCGCGAGAATACTAAC
nit-1 (qRT-PCR) forward	AATCCTCCGACTATCCCTTG
nit-1 (qRT-PCR) reverse	AGCGAATCGTTTCTTTTGTG
sip-1 (qRT-PCR) forward	AAGAGATCGTTCACTCGCCAG
sip-1 (qRT-PCR) reverse	AGCCAAGTCGACGTCCTTTG
hsp-16.2 (qRT-PCR) forward	CTCTGATGGAACGCCAATTT
hsp-16.2 (qRT-PCR) reverse	ACGTTGAGATTGATGGCAAA
hsp-70 (qRT-PCR) forward	TCATGCAAAGCTATTGGTATCG
hsp-70 (qRT-PCR) reverse	TGCCACGTATGATGGAGTTG
cth-2 (qRT-PCR) forward	TTGGAGCGGATGTTGTCGTT
cth-2 (qRT-PCR) reverse	CGACGGCAAGTTGCATGAAG
SR124 (atf-4(tm4397) forward	TCTGAAAATTATGAACTTCAACATGA
SR125 (atf-4(tm4397) reverse	TGAGTTGAACTTTCAAACGGAGT
RV103_cth-2(mg599) forward	TTCATTGAAACTTTCGGCGTG
RV104_cth-2(mg599) reverse	ACATCCGCTCCAAGAGAAATT
RV105_cth-2(mg599) seq	GTCCTGAAGAGAACACTTGGC

Supplementary References

1 Fiorese, C. J. *et al.* The Transcription Factor ATF5 Mediates a Mammalian Mitochondrial UPR. *Curr Biol* **26**, 2037-2043, doi:10.1016/j.cub.2016.06.002 (2016).

2 Chen, J. *et al.* Pervasive functional translation of noncanonical human open reading frames. *Science* **367**, 1140-1146, doi:10.1126/science.aay0262 (2020).

3 Park, Y., Reyna-Neyra, A., Philippe, L. & Thoreen, C. C. mTORC1 Balances Cellular Amino Acid Supply with Demand for Protein Synthesis through Post-transcriptional Control of ATF4. *Cell Rep* **19**, 1083-1090, doi:10.1016/j.celrep.2017.04.042 (2017).

4 Costa-Mattioli, M. & Walter, P. The integrated stress response: From mechanism to disease. *Science* **368**, doi:10.1126/science.aat5314 (2020).

5 Robida-Stubbs, S. *et al.* TOR signaling and rapamycin influence longevity by regulating SKN-1/Nrf and DAF-16/FoxO. *Cell Metab* **15**, 713-724, doi:10.1016/j.cmet.2012.04.007 (2012).
