# Supplementary material for: ATF-4 and hydrogen sulfide signalling mediate longevity in response to inhibition of translation or mTORC1
Source: Nat Commun. 2022 Feb 18;13:967. doi: 10.1038/s41467-022-28599-9 (PMC8857226; doi:10.1038/s41467-022-28599-9)
Supplement: Supplementary file 3 — Description of Additional Supplementary Files [file 41467_2022_28599_MOESM3_ESM.pdf]

Description of Additional Supplementary Files

File Name: Supplementary Data 1
Description: Overexpression of ATF-4 increases lifespan

File Name: Supplementary Data 2
Description: Pumping rates measured under different conditions. Columns A-J: individual C. elegans pharyngeal pumping rate (pumps/min) on food.

File Name: Supplementary Data 3
Description: RNA sequencing of ATF4oe vs wild type vs *atf-4(mutants)*

File Name: Supplementary Data 4
Description: Supplementary Data 4: Summary of potential shared direct ATF4 target genes between mammals and *C. elegans*

File Name: Supplementary Data 5
Description: Venn diagrams of upregulated genes upon *ldIs119* ATF-4 overexpression with DAF-16, HSF-1, SKN-1

File Name: Supplementary Data 6
Description: Supplementary Data 6: High Performance Liquid Chromatography (HPLC) comparing ATF4 overexpressor vs wild type

File Name: Supplementary Data 7
Description: Thermotolerance assays either manually or with automated lifespan machine

File Name: Supplementary Data 8
Description: Arsenite oxidative stress assays.

File Name: Supplementary Data 9
Description: Patf-4GFP scoring

File Name: Supplementary Data 10
Description: Oxidative stress assays t-BOOH summary and raw data.

File Name: Supplementary Data 11
Description: CTH mRNA levels in different tissues under different conditions.

File Name: Supplementary Data 12
Description: Quantification of H2S assays
