# Supplementary material for: ATF-4 and hydrogen sulfide signalling mediate longevity in response to inhibition of translation or mTORC1
Source: Nat Commun. 2022 Feb 18;13:967. doi: 10.1038/s41467-022-28599-9 (PMC8857226; doi:10.1038/s41467-022-28599-9)
Supplement: Supplementary file 16 — Reporting Summary [file 41467_2022_28599_MOESM16_ESM.pdf]

nature research

Corresponding author(s): Collin Ewald, T. Keith Blackwell

Last updated by author(s): 12/15/2021

Reporting Summary

Nature Research wishes to improve the reproducibility of the work that we publish. This form provides structure for consistency and transparency in reporting. For further information on Nature Research policies, see Authors & Referees and the Editorial Policy Checklist.

Statistics

For all statistical analyses, confirm that the following items are present in the figure legend, table legend, main text, or Methods section.

n/a	Confirmed	
☐	☒	The exact sample size (*n*) for each experimental group/condition, given as a discrete number and unit of measurement
☐	☒	A statement on whether measurements were taken from distinct samples or whether the same sample was measured repeatedly
☐	☒	The statistical test(s) used AND whether they are one- or two-sided<br>*Only common tests should be described solely by name; describe more complex techniques in the Methods section.*
☒	☐	A description of all covariates tested
☐	☒	A description of any assumptions or corrections, such as tests of normality and adjustment for multiple comparisons
☐	☒	A full description of the statistical parameters including central tendency (e.g. means) or other basic estimates (e.g. regression coefficient) AND variation (e.g. standard deviation) or associated estimates of uncertainty (e.g. confidence intervals)
☐	☒	For null hypothesis testing, the test statistic (e.g. *F*, *t*, *r*) with confidence intervals, effect sizes, degrees of freedom and *P* value noted<br>*Give P values as exact values whenever suitable.*
☒	☐	For Bayesian analysis, information on the choice of priors and Markov chain Monte Carlo settings
☒	☐	For hierarchical and complex designs, identification of the appropriate level for tests and full reporting of outcomes
☒	☐	Estimates of effect sizes (e.g. Cohen's *d*, Pearson's *r*), indicating how they were calculated

Our web collection on statistics for biologists contains articles on many of the points above.

Software and code

Policy information about availability of computer code

Data collection	www.wormbase.org (WS258). Ribosome profiling sequencing data were downloaded from the NCBI Sequence Read Archive (S.R.A.) (http://www.ncbi.nlm.nih.gov/sra/) under accession number SRA055804. Microarray datasets and platform information were obtained from GEO (https://www.ncbi.nlm.nih.gov/geo/) and SRA (https://www.ncbi.nlm.nih.gov/sra).
Data analysis	Exon-Intron Graphic Maker (http://wormweb.org/exonintron). ApE- A plasmid Editor v2.0.50b3. For amino acid alignments T-COFFEE (Version_11.00.d625267). FastQC, FASTQ, SAMtools (version 0.1.19), JMP software v.9.0.2., Prism 6.0 software (GraphPad), STAR 2.4.0j software, Bioconductor (http://bioconductor.org), Rsubread 1.16.1 featureCounts, Degust (http://degust.erc.monash.edu), edgeR, limma, Ortholist2, Trim Galore (https://www.bioinformatics.babraham.ac.uk/projects/trim_galore/), Salmon, survminer (https://rpkgs.datanovia.com/survminer/), ggplot2 (https://ggplot2.tidyverse.org, version 3.3.5) R package, dplyr (https://dplyr.tidyverse.org/reference/dplyr-package.html, version 1.0.7) R package.

For manuscripts utilizing custom algorithms or software that are central to the research but not yet described in published literature, software must be made available to editors/reviewers. We strongly encourage code deposition in a community repository (e.g. GitHub). See the Nature Research guidelines for submitting code & software for further information.

Data

Policy information about availability of data

All manuscripts must include a data availability statement. This statement should provide the following information, where applicable:

- Accession codes, unique identifiers, or web links for publicly available datasets
- A list of figures that have associated raw data
- A description of any restrictions on data availability

The RNA sequencing data in this publication have been deposited in NCBI's Gene Expression Omnibus and are accessible through GEO Series accession number GSE173799 [https://www-ncbi-nlm-nih-gov.ezproxy.u-pec.fr/geo/query/acc.cgi?acc=GSE173799].
Source data are provided with this paper. Supplementary Table 1 is the raw data for Fig 1g, 1h, 2a, 2f, Sup Fig 2c, 3g, 4a, 4b, 4c, 4e, 4i, 5d, Sup fig 5d, Sup fig 5e, and 6a. Supplementary Table 2 is the raw data for Fig 2b and Sup Fig 2c. Supplementary Table 3 is the raw data for Fig 2c, Sup Fig 3c, and Sup Fig 3d. Supplementary

Table 4 is the raw data for Fig 2d. Supplementary Table 5 is the raw data for Sup fig 3h. Supplementary Table 7 is the raw data for Fig 3h, 4h, and 5c. Supplementary Table 8 is the raw data for Sup Fig 4f and Sup Fig 4g. Supplementary Table 9 is the raw data for Fig 4d, Sup Fig 5b, and Sup Fig 5c. Supplementary Table 10 is the raw data for Fig 4g.
Ribosome profiling sequencing data were downloaded from the NCBI Sequence Read Archive (S.R.A.) (http://www.ncbi.nlm.nih.gov/sra/) under accession number SRA055804.

Field-specific reporting

Please select the one below that is the best fit for your research. If you are not sure, read the appropriate sections before making your selection.

☒ Life sciences ☐ Behavioural & social sciences ☐ Ecological, evolutionary & environmental sciences

For a reference copy of the document with all sections, see nature.com/documents/nr-reporting-summary-flat.pdf

Life sciences study design

All studies must disclose on these points even when the disclosure is negative.

Sample size	No statistical methods were used in choosing sample sizes. Instead, all sample sizes were chosen based on standard C.elegans technique protocols published before, except to measure endogenous H2S levels and PSSH levels.<br>For all WB experiments and qRT-PCR experiments, at least 3 independent samples were prepared, which is common in this field (Chamoli et al., Nat Commun, 2020; Topf et al., Nat Commun, 2018; Wu et al., Cell Metabolism, 2019; Su et al., Nat Commun, 2019).<br>For lead acetate assays, at least 2 independent experiments were conducted, and various concentrations of protein lysates were analyzed, which is common in the field (Wei and Kenyon, PNAS, 2016; Hine et al., Cell, 2015).<br>For fluorescent reporter quantification, at least 2 independent experiments containing a total of at least 30 worms were conducted (Ewald et al., eLife, 2017; Meng et al., Nat Commun, 2021).<br>For lifespan and stress assays, at least 60-100 or 30-60 animals were examined per condition, respectively, which is common in the field (Meng et al., Nat Commun, 2021; Wu et al., Cell Metabolism, 2019; Ewald et al., eLife, 2017).<br>To measure endogenous H2S levels and PSSH levels, at least 10 worms over 3 biological replicates were examined per condition/genotype. These assays are newly developed and very sensitive.
Data exclusions	No data exclusion.
Replication	The number of biological independent repeats is indicated in the figure legends and supplemental figures. No attempts to replicate failed.
Randomization	C. elegans were randomly allocated into experimental groups.
Blinding	In analyses of fluorescent reporters, either all or representative trials were scored blindly.<br>Western blot images, qRT-PCR, RNA sequencing, and ribosomal profiling data were obtained and analyzed by commercial software. Blinding is not typically done for such assays.<br>Automated survival assays were performed by machines.<br>For manual survival analyses and other fluorescence microscopy assays, investigators were careful to maintain objectivity and used clearly defined scoring criteria (mentioned in Methods).

Reporting for specific materials, systems and methods

We require information from authors about some types of materials, experimental systems and methods used in many studies. Here, indicate whether each material, system or method listed is relevant to your study. If you are not sure if a list item applies to your research, read the appropriate section before selecting a response.

Materials & experimental systems

n/a	Involved in the study	
☐	☒	Antibodies
☒	☐	Eukaryotic cell lines
☒	☐	Palaeontology
☐	☒	Animals and other organisms
☒	☐	Human research participants
☒	☐	Clinical data

Methods

n/a	Involved in the study	
☒	☐	ChIP-seq
☒	☐	Flow cytometry
☒	☐	MRI-based neuroimaging

Antibodies

Antibodies used	Tubulin (1:500, Sigma #T9026), Puromycin (1:10’000, Millipore #MABE343), GFP (1:1’000, Roche #11814460001), Cystathionase/CTH (1:2000, abcam #ab151769) and Phospho-eIF2alpha (Ser51) (1:1’000, CellSignal #9721). HRP-conjugated goat anti-mouse (1:2’000, Cell Signaling #7076) and goat anti-rabbit (1:2’000, Cell Signaling #7074) secondary antibodies were used.

Validation	Specificity of Cystathionase/CTH (1:2000, abcam #ab151769) was confirmed by cth-2(RNAi).<br>Tubulin, p-eif2a antibodies recognize evolutionarily conserved sites and are routinely used in C.elegans. Publications that have used these antibodies include: Meng et al., Nat Commun, 2021; Ewald et al., eLife, 2017; Derisbourg et al., Nat Commun, 2021.<br>Puromycin antibody was validated by a previous paper (Derisbourg et al., Nat Commun, 2021).

Animals and other organisms

Policy information about studies involving animals; ARRIVE guidelines recommended for reporting animal research

Laboratory animals	Caenorhabditis elegans strains are listed in supplementary table 13. All animals used were hermaphrodites. Lifespan and pumping rate assays were performed during the whole life of animals. For all other assays, the stage of animals are stated in figure legends and Methods.
Wild animals	No wild animals were used in this study.
Field-collected samples	No filed-collected samples were used in this study.
Ethics oversight	No ethical approval or guidance was required for C.elegans.

Note that full information on the approval of the study protocol must also be provided in the manuscript.
